# Revisiting Hunter Perceptions toward Chronic Wasting Disease: Changes in Behavior over Time

**DOI:** 10.3390/ani10020187

**Published:** 2020-01-22

**Authors:** Angela M. Holland, Jacob M. Haus, T. Brian Eyler, Mark D. Duda, Jacob L. Bowman

**Affiliations:** 1 Department of Entomology and Wildlife Ecology, University of Delaware, 531 S College Avenue, Newark, DE 19716, USA; 2 Department of Biology, Bemidji State University, 1500 Birchmont Drive, Bemidji, MN 56601, USA; 3 Maryland Department of Natural Resources, 14038 Blairs Valley Road, Clear Spring7, MD 21722, USA; 4 Responsive Management, 130 Franklin Street, Harrisonburg, VA 22801, USA

**Keywords:** *cervus nippon*, chronic wasting disease, harvest, hunter perception, *Odocoileus virginianus*, sika deer, white-tailed deer

## Abstract

**Simple Summary:**

Hunters play a vital role in the management of wildlife diseases such as Chronic Wasting Disease, but their harvest rates may change based on their perception of disease risk. Our objective was to estimate how hunter harvest may change over time based on perception of disease and proximity to disease location. We found that hunters harvested fewer deer in the 4 years following disease discovery but that in the next 4-year period harvest rates increased to be similar to those from before the discovery of the disease. This indicates that changes in behavior due to disease presence may diminish over time. Understanding how hunters’ change their behavior in relation to disease presence will aid wildlife managers in creating plans to manage wildlife populations and diseases.

**Abstract:**

Hunter behavior varies in relation to perceived risk of Chronic Wasting Disease (CWD) and changes in perceptions of CWD will lead to changes in behavior over time. During 2018, we surveyed deer (*Odocoileus virginianus* or *Cervus nippon*) hunters from Maryland, USA, regarding behavioral changes due to CWD. We matched 477 respondents to their harvest record and created two geographical groups based on harvest history in counties closest to disease presence. We compared the proportion of hunters who claimed to have changed their behavior in each group and estimated the effects of CWD on harvest rate for the 4 years immediately after the discovery of CWD and the following 4-year period. We found no difference between the groups in the proportion of hunters who changed their behavior due to CWD. We found a significant decline in harvest rate for hunters who claimed to change their behavior in the group closest to CWD presence during the period immediately after the discovery of CWD; however, these same hunters increased their harvest rates in the next time period to pre-CWD levels. Overall, we found that time alleviates some perceived risk of CWD and that this is reflected in hunting behavior.

## 1. Introduction

The fatal neurological disease, Chronic Wasting Disease (CWD), is known to affect deer (*Odocoileus* spp.), Rocky Mountain elk (*Cervus canadensis nelsoni*), moose (*Alces alces*), reindeer (*Rangifer tarandus*) and may affect other cervids [1,2,3,4,5]. The spread and management of this or any disease which affects game species is a primary concern for state biologists and stakeholders [6]. CWD is spread through contact with contaminated material; therefore, transmission rates are likely greater in areas with higher cervid densities and access to artificial bait [4,6]. To manage spread of CWD, legislation enacted may be directly related to harvest regulations (e.g., increased harvest limits), bans on artificial feeding, or carcass translocation [6,7]. Additionally, management agencies may practice selective culling [8]. When using increased harvest limits to decrease population sizes, success is dependent on hunter participation which in turn is dependent on hunter perceptions of CWD and CWD management [9,10].

Hunters vary in their perception of the risk CWD poses to their own health and their future hunting opportunities. Those who are the most risk sensitive (moderate risk) are more likely to claim that they altered their hunting behavior than hunters with slight or no perceived risk [11]. This risk-behavior relationship may be due to fear that reduced populations for disease control may not be effective and only result in fewer harvest opportunities in the future or fear of contracting the human variant of the disease, Creutzfelt-Jakob disease [12,13,14]. Altered behavior may include reduced hunter participation in CWD areas (e.g., decreased license sales, reduced time spent hunting, change of hunting location) and result in an inability to reduce deer populations and thus the spread of disease, or revenue loss [9,10]. These changes in hunter behavior due to perceptions of the disease will weaken the effect recreational hunting has on CWD management and should be of concern to wildlife managers.

Most research examining hunter perceptions of CWD involves theoretical behavioral responses [12,14,15,16] and few studies have examined actual changes in hunter participation [9,10]. Previous research on how hunter perceptions affect actual harvest behavior in CWD-infected areas found decreased rates of harvest in the 2 years post CWD discovery [10]. This decrease in harvest only occurred for hunters with negative perceptions of CWD who were hunting in the area where CWD was discovered [10]. This differs from research on risk perceptions which indicate hunters closer to areas with CWD have lower perceived risk than those farther away [17]. Over time, hunter perceptions of CWD are likely to change. This change may be due to increased perceived risk of the disease as its prevalence increases [9,16] or perceived risk may decrease with experience with the disease [18].

Changes in hunter perceptions of CWD will lead to changes in behavior over time. Using previous research in Maryland as our study template [10], we revisited hypotheses regarding changes in behavior as reported by the hunter and changes in hunter harvest due to CWD discovery and expansion. We hypothesized that the proportion of hunters with changed behavior due to CWD would be greater in areas in or near the CWD Management Area (CWDMA). We also hypothesized that hunter harvest would be lower for hunters with reported changed behavior in or near the CWDMA for a short time period after CWD discovery, similar to previous research [10]. Additionally, we hypothesized that after this short time period, harvest rates of these same hunters would increase due to decreased risk perception [18].

## 2. Materials and Methods

### 2.1. History of CWD in Study Area

**Figure 1:**
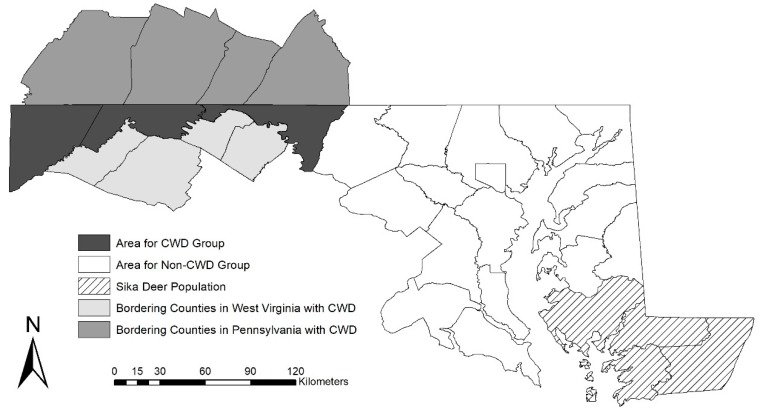
Location of counties for the Chronic Wasting Disease (CWD) Group and Non-CWD Group that survey respondents were divided into based on where they had harvested deer from 2008–2019 in Maryland, USA and the location of the sika deer population and counties from neighboring states with CWD.

Maryland Department of Natural Resources (MDNR) first discovered CWD in Maryland, USA in 2010 when a 1.5-year-old male white-tailed deer (*O. virginianus*) harvested in southern Allegany County tested positive for the disease [19]. MDNR established a CWDMA in the eastern portion of Allegany County after the discovery of CWD and placed restrictions on carcass translocation and artificial feeding within the area [19]. By 2015, MDNR discovered 10 additional cases of CWD in Allegany County, all but one of these was inside of the CWDMA. The CWDMA was expanded in 2016 to include all of Allegany County and western Washington County [19]. Sixteen more white-tailed deer tested positive for CWD by the end of the 2017–2018 hunting season, all were harvested within the expanded CWDMA [20]. All positive cases were free-ranging white-tailed deer. Maryland also had a population of sika deer (*Cervus nippon*) located on the eastern shore of the Chesapeake Bay (Figure 1). This population was not near the CWDMA nor are sika deer known to contract CWD, however as members of the Cervidae family, are likely susceptible. West Virginia was the first state in the region to detect the disease which is currently found in West Virginia, Maryland, Pennsylvania, and Virginia, USA. West Virginia had 376 reported cases of CWD in white-tailed deer from 2005–2019 with the majority (94%) from counties bordering Allegany County, Maryland [21]. Pennsylvania reported CWD in 283 deer from 2012–2019, 75% of these reports were from counties bordering Allegany County, Maryland [22].

### 2.2. Population Sampled

Responsive Management (Harrisonburg, VA, USA) conducted a survey of Maryland residents, landowners, and hunters regarding attitudes toward deer management and hunting during July 2018 [23]. The survey was ruled exempt from review by the University of Delaware Institutional Review Board due to federal regulations. Responsive Management conducted surveys by telephone based on questionnaires. To avoid bias toward people easily reached by telephone, a five-callback design was used to reach respondents. They conducted surveys Monday through Friday noon to 9:00 pm, Saturday from noon to 5:00 pm and Sunday from 5:00 pm to 9:00 pm. Respondents included the general population (800 completed surveys), landowners with 8.1 ha or more of land and that grew agricultural crops (606 completed surveys), and deer hunters who purchased a Maryland hunting license for the 2017–2018 hunting season (801 completed surveys) [23] with an overall response rate of 26%. We focus only on responses from the deer hunters.

We used hunter ID numbers to match hunter survey responses to harvest data collected by the state of Maryland starting with the 2008–2009 hunting season. We only included hunters that had at least a 10-year hunting record as indicated by either purchasing a hunting license or harvesting a deer for the 2008–2009 or 2009–2010 hunting seasons (private landowners are not required to purchase licenses in the state of Maryland). We excluded hunters who did not harvest any deer from 2008 through the 2018–2019 hunting season. Hunters who harvested at least one deer (white-tailed deer or sika deer) in the 11-year period were grouped into those who harvested deer in counties affected by or adjacent to the CWDMA (Allegany, Garrett, and Washington Counties) and all other Maryland counties (Figure 1). Hereafter these groups are referred to as CWD Group and Non-CWD Group. If hunters harvested deer in one of the CWD counties and elsewhere in Maryland they were put into the CWD Group. Deer harvest bag limits did not change over the study period and all harvested deer were required to be registered.

### 2.3. Survey Analysis

We only used a subset of questions and attitudinal measures related to CWD. Surveyors asked respondents, “Prior to this survey, had you heard of Chronic Wasting Disease or CWD?”. We removed any hunter who answered “No” or “I don’t know” from our sample population (*n* = 477; CWD Group = 124, Non-CWD Group = 353) to limit our inference to hunters who were aware of CWD and had the choice to change their hunting behavior. We used 2 perception statements about hunter behavior: (1) CWD has caused you to change where you hunt deer in Maryland; and (2) CWD has caused you to hunt deer less often in Maryland in general. Respondents indicated if they “strongly agreed”, “moderately agreed”, “neither agreed nor disagreed”, “moderately disagreed”, “strongly disagreed”, or “don’t know” with these statements.

We tested for differences in attitude toward CWD between hunters in the CWD Group and Non-CWD Group by differentiating between those who claimed CWD had changed their hunting behavior and those who did not. Negative hunters indicated they “strongly agreed” or “moderately agreed”, while non-negative hunters “strongly disagreed”, “moderately disagreed”, or “neither agreed nor disagreed”. We removed respondents who answered “don’t know” from analysis for the corresponding statement. We used a chi-square test of independence to determine differences in attitudes of hunters in the CWD Group or Non-CWD Group.

We evaluated changes in harvest rate for hunters in the CWD Group and Non-CWD Group over the 11-year period using the reported harvest data associated with each hunter ID number. We divided the harvest history into 3 periods: pre-CWD (2008–2009, 2009–2010, and 2010–2011), immediately post-CWD (2011–2012, 2012–2013, 2013–2014, and 2014–2015), and 4-years post-CWD (2015–2016, 2016–2017, 2017–2018, and 2018–2019). We calculated harvest rates for all hunters for each period using the average number of deer harvested/individual/year. If a hunter harvested deer in both areas, and was therefore only included in the CWD Group, we only used the deer they harvested in Allegany, Garrett, and Washington Counties to calculate the average harvest rate. For this aspect of the analysis hunters were considered negative if they answered, “strongly agree” or “moderately agree” to either statement and non-negative if they answered, “strongly disagree”, “moderately disagree”, or “neither agree nor disagree” to both statements (*n* = 474; CWD Group = 122, Non-CWD Group = 352). We tested for differences in average harvest rate between pre-CWD and immediately post-CWD, as well as, between pre-CWD and 4-years post-CWD using paired t-tests for negative and non-negative hunters in both areas. We used Bonferroni corrections for the critical alpha value and measured effect size using Cohen’s D. Cohen’s D evaluates effect size based on standard deviation with D ≥ 0.2 equating to a small effect size, ≥ 0.5 a medium effect size, and ≥ 0.8 a large effect size [24]. All analyses were conducted using Program R version 3.6.1 [25].

Following the methods of Haus et al. [10], we calculated change in average harvest rates between pre-and immediately post-CWD, as well as, pre- and 4-years post-CWD. We used the difference in change of harvest rate for non-negative hunters and change of harvest rate for negative hunters to generate ∆harvest for the CWD Group and Non-CWD Group:

∆harvest = (a − b) − (x − y),(1)

where a = the average harvest rate for negative hunters pre-CWD, b = the average harvest rate for negative hunters immediately post-CWD or 4-years post-CWD, x = the average harvest rate for non-negative hunters pre-CWD, and y = the average harvest rate for non-negative hunters immediately post-CWD or 4-years post-CWD. ∆harvest was the post-CWD reduction in potential deer harvest rates among negative hunters relative to non-negative hunters. We assumed we could attribute ∆harvest to changed behavior due to CWD and management regulations and not to stochastic variation in harvest (i.e., weather variables, mast abundance, deer abundance). We then used the total number of hunters who had registered a deer from fall 2011 to winter 2015 and fall 2015 to winter 2019, the percent of negative respondents, and ∆harvest to extrapolate the average annual reduction in potential harvest for each area for each post-CWD time period;

R = −∆harvest × (H × h),(2)

where R = the average annual reduction in harvest related to CWD, H = the total number of hunters who registered a deer during each post discovery time period, and h = the percentage of hunters with changed behavior due to CWD.

## 3. Results

A relatively small percentage of respondents to the 2018 survey (11.7%) claimed to have altered their hunting behavior due to CWD. Most of these respondents claimed to have changed where they hunt in Maryland (92.9% of 56 respondents) and fewer respondents indicated that they hunted less (32.1%). Only 2.9% of the 477 respondents indicated that they changed their behavior by changing where they hunt and hunting less. Proportionally more hunters in the CWD Group reported to change where they hunted or hunt less due to CWD than hunters in the Non-CWD Group; however, there was no significant difference between the two groups (Table 1).

We investigated how harvest rates changed over time for negative and non-negative attitudes by comparing average annual harvest rate before, immediately after, and 4-years post CWD discovery. Harvest rates were similar between non-negative and negative hunters in the CWD Group prior to the discovery of CWD (0.78 and 0.79 respectively). In the period immediately after the discovery of CWD, harvest rates of negative hunters in the CWD Group dropped significantly and had a medium effect size using Cohen’s D (Table 2). Harvest rates of negative hunters in the CWD Group recovered to rates similar to those prior to the discovery of CWD by the 4-year post CWD period (Table 3). Non-negative hunters in the CWD Group maintained stable harvest rates throughout all three periods (Table 2 and Table 3). The ∆harvest was lower for the 4-year post CWD period indicating that the negative effects of CWD discovery on hunter harvest have decreased over time (Table 3). Across the three county CWD area, the relative decrease in deer harvest due to CWD was 0.174 deer/km^2^/year immediately following CWD discovery (Table 2), but only 0.039 deer/km^2^/year in the 4-year post discovery time period (Table 3). In comparison to the yearly average harvest for this area over the 3 years before CWD discovery, this is a 4.1% and 0.9% reduction in deer harvest due to CWD [26,27,28].

Non-negative hunters in the Non-CWD Group had greater rates of harvest than negative hunters prior to the discovery of CWD (1.45 and 1.12, respectively). Harvest rates of negative hunters stayed relatively stable throughout all three periods (Table 2 and Table 3), but harvest rates for non-negative hunters dropped over time. This decrease in harvest rate was significant between the pre-CWD period and the 4-year post CWD period (*p* = 0.001), however the Cohen’s D was less than 0.2 indicating that the difference was trivial (Table 3). Harvest rates of negative and non-negative hunters in the Non-CWD Group were similar in the 4-year post CWD period (1.17 and 1.12, respectively). Because harvest rates for negative hunters remained stable over the study period but harvest rates for non-negative hunters decreased, the ∆harvest was negative for both time period comparisons in the Non-CWD Group. This finding translates to an increase in harvest related to CWD and when extrapolated, resulted in an increase of 0.075 deer/km^2^/year immediately post CWD discovery and 0.100 deer/km^2^/year 4-years-post CWD discovery in Maryland outside of the tri-county CWD area. This equates to a 2.4% and 3.2% increase in deer harvest for this area in comparison to the average deer harvest for the 3-year period before the discovery of CWD [26,27,28].

## 4. Discussion

Decreases in harvest rates due to the discovery of CWD appear to be temporary and return to pre-CWD discovery levels over time. Negative hunters in the CWD Group had lower harvest rates in the 4-years immediately following the discovery of CWD; however, their harvest rates increased in the next 4-year time period. These findings support two of our hypotheses. We did not find support for our hypothesis that the proportion of hunters with reported changed behavior due to CWD would be higher in areas near the CWDMA.

Similar to Haus et al. [10], we found that negative hunters, hunting in the CWD discovery area, had a significant decrease in harvest rates after the discovery of CWD. This was the only pairwise comparison that had both a statistically significant decline and a non-trivial effect size. The average harvest rate for the pre-CWD period was greater among our survey respondents (0.78) than the 2013 survey respondents (0.62 for Allegany County), but the harvest rate immediately post-CWD discovery was very similar (0.46 and 0.47; [10]). This similarity occurred despite a 2-year difference in the time period over which the harvest rate was averaged and an expanded geographic area.

In the CWD Group, hunter harvest rates increased in the 4-year post-CWD discovery time period to levels similar to harvest rates before CWD discovery. This finding supported our hypothesis that hunter harvest rates would increase over time due to decreases in risk perception of CWD. While we did not explicitly test for changes in hunters risk perception, previous research indicates that perceived risk decreases with time [18,29] and our findings demonstrate that hunting behavior reflects these changes in perceived risk. At the time of the survey in 2018, a total of 27 cases of CWD had been identified, all within the CWDMA expanded in 2016. The number of CWD cases in Maryland more than doubled between 2016 and 2018. In 2019, 25 additional cases were discovered and the CWDMA area was again expanded to include all of Washington, as well as Allegany County. This increase in the rate of positive tests for deer with CWD may lead to future changes in CWD perceptions and hunter harvest for hunters in the CWDMA and nearby areas.

We hypothesized that the proportion of hunters with reported changed behavior due to CWD would be greater in areas near the CWDMA since differences were found in previous research [10]; however we did not find statistical differences in the proportion of negative hunters between the CWD Group and the Non-CWD Group for either perception statement. Our inability to replicate the result of Haus et al. [10] could be due to sample size, time elapsed since the discovery of CWD, the inclusion of counties overlapping and near to the CWDMA, or a lack of repeatability. Of these options, one or a combination of the first three are most likely. We did have a greater proportion of negative hunters in the CWD Group in comparison to the Non-CWD Group, but we had a smaller sample size than Haus et al. [10], which may have resulted in an inability to detect a significant difference.

Our survey was conducted 8 years after the discovery of CWD, while the previous survey occurred 3 years after. This difference in time may have resulted in a decrease in the proportion of negative hunters in the CWD Group. Over time, perceptions of disease risk for CWD have decreased even in states with high levels of prevalence [18,29]. Hunters taking the 2013 survey may have been more likely to indicate that they had changed their behavior after the discovery of CWD than those taking the 2018 survey because CWD discovery was novel and changes they had made or planned to make were recent. Furthermore, hunters taking the 2018 survey may have forgotten small behavioral changes they made after the discovery of CWD and did not indicate that they had changed their behavior.

We combined hunters in Allegany, Garrett, and Washington Counties since these counties were part of or bordering the expanding CWDMA, while Haus et al. [10] examined Allegany, Garrett, and a county away from the CWDMA separately (Dorchester County). Significantly more of the Allegany County respondents indicated that they changed where they hunt in the 2013 survey than hunters in Garrett or Dorchester Counties [10]. By pooling hunters in the three counties most likely to be affected by CWD, we may have decreased the proportion of hunters who responded negatively to each statement. In the 2013 survey, 15.9% of hunters from Allegany County indicated that they changed where they hunt and 10.2% of Garrett County hunters changed their hunting location [10]. If these hunters are pooled, 13.7% of respondents in these two counties indicated that they changed where they hunt deer in Maryland. This value is close to the proportion of hunters who indicated a change in where they hunt deer in the 2018 survey, 14.8%. The proportion of hunters in the Non-CWD Group who indicated a change in where they hunt (9.6%) was also similar to the proportion of hunters in Dorchester County who indicated the same change in behavior in 2013 (9.7%). This supports our conclusion that we were unable to find differences in the proportion of hunters in the CWD Group and Non-CWD Group who changed where they hunt because we combined multiple counties with slightly different CWD histories and also potentially because of a small sample size.

The estimated relative reduction in harvest due to CWD in the tri-county area closest to the CWDMA during the 4-year period immediately post-CWD discovery was 4.1% which was lower than the 7.0% estimated relative reduction in Allegany County after the 2013 survey [10]. This reduction is within the normal stochastic variation in harvest for these counties prior to CWD discovery [26,27,28]. The estimated relative change in harvest due to CWD in the Non-CWD Group was an increase in harvest since harvest rates for negative hunters remained stable while harvest rates for non-negative hunters decreased for both post-CWD periods in comparison to the pre-CWD period. The decrease in harvest rate for non-negative hunters is similar to declines in total harvest for the state of Maryland. Average annual harvest for the state declined by 7.9% and 16.7% for the periods immediately post-CWD discovery and 4-years post-CWD discovery, respectively, in comparison to the 3 years prior to CWD discovery [20,26,27,28,30,31,32,33,34,35,36]. These statewide declines cannot be attributed to changes in hunting behavior due to CWD since the pattern is best represented by the group of hunters who claimed that they did not change their behavior due to CWD and were farther from the CWDMA.

## 5. Conclusions

Our findings support previous research indicating that changes in hunting behavior due to the presence of CWD are local to the geographical area with the infected deer population and have minimal impact on overall deer harvest [10]. Reductions in deer harvest rates due to CWD were within the annual stochastic variation for the counties where CWD was found and only occurred for a short period of time (approximately 4 years). We recognize that our study does not account for hunters who completely stopped hunting after the discovery of CWD since survey respondents purchased a hunting license for the 2017–2018 hunting season. However previous research indicates that even hunters who claimed to stop hunting after the discovery of CWD continued to do so [10]. Increasing prevalence of the disease complicates the ability to accurately predict hunter behavior over long periods of time, but after 8 years of CWD presence in Maryland harvest rates in the counties closest to the CWDMA returned to pre-CWD levels for our surveyed hunters indicating that time alleviates some perceived risk and that this is reflected in hunting behavior.

## Figures and Tables

**Table 1:** Respondents who either agreed or strongly agreed to statements regarding behavioral changes in response to Chronic Wasting Disease (CWD) in a survey of hunters in Maryland, USA, during 2018. *n* is the total sample including all hunter respondents.

CWD Has Caused You to	Total (*n*)	CWD Group (*n*)	Non-CWD Group (*n*)	Χ^2^	*p*-Value
change where you hunt deer in Maryland	10.95% (475)	14.75% (122)	9.63% (353)	2.440	0.118
hunt less in Maryland in general	3.78% (476)	5.65% (124)	3.13% (352)	1.601	0.206

**Table 2:** Harvest rates (deer/hunter/year) for hunters with negative and non-negative attitudes toward Chronic Wasting Disease (CWD) before (2008–2011) and immediately after CWD discovery (2011–2015) and the associated changes in harvest for the CWD Group and Non-CWD Group based on proximity to the disease management area in Maryland, USA, during 2018. *p* < 0.025 indicated by *.

Variable	CWD Group	Non-CWD Group
Negative Hunters		
Pre-CWD average harvest rate	0.778	1.123
Immediately Post-CWD average harvest rate	0.461	1.273
Difference in harvest Pre to Immediately Post	0.317	−0.150
Paired *t*-test *p*-value (t, DF)	0.007 * (3.0849, 17)	0.299 (−1.054, 37)
Cohen’s D	0.563	−0.132
**Non-Negative Hunters**	**CWD Group**	**Non-CWD Group**
Pre-CWD average harvest rate	0.785	1.452
Immediately Post-CWD average harvest rate	0.734	1.327
Difference in harvest Pre to Immediately Post	0.051	0.125
Paired *t*-test *p*-value (t, DF)	0.797 (−0.258, 103)	0.922 (0.098, 313)
Cohen’s D	−0.024	0.004
**Change Due to CWD**	**CWD Group**	**Non-CWD Group**
Δ harvest due to CWD	0.266	−0.275
Change in annual harvest due to CWD by km^2^	−0.174	0.075

**Table 3:** Harvest rates (deer/hunter/year) for hunters with negative and non-negative attitudes toward Chronic Wasting Disease (CWD) before (2008–2011) and 4-years after CWD discovery (2015–2019) and the associated changes in harvest for the CWD Group and Non-CWD Group based on proximity to the disease management area in Maryland, USA, during 2018. *p* < 0.025 indicated by *.

Variable	CWD Group	Non-CWD Group
Negative Hunters		
Pre-CWD average harvest rate	0.778	1.123
4 years Post-CWD average harvest rate	0.663	1.172
Difference in harvest Pre to 4 years Post	0.115	−0.049
Paired *t*-test *p*-value (t, DF)	0.743 (0.334, 17)	0.530 (−0.634, 37)
Cohen’s D	0.079	−0.067
**Non-Negative Hunters**	**CWD Group**	**Non-CWD Group**
Pre-CWD average harvest rate	0.785	1.452
4 years Post-CWD average harvest rate	0.730	1.122
Difference in harvest Pre to 4 years Post	0.055	0.330
Paired *t*-test *p*-value (t, DF)	0.993 (−0.008, 103)	0.001 * (3.321, 313)
Cohen’s D	−0.001	0.171
**Change Due to CWD**	**CWD Group**	**Non-CWD Group**
∆harvest due to CWD	0.060	−0.379
Change in annual harvest due to CWD by km^2^	−0.039	0.100

## References

[1] Williams E.S., Young S. (1980). Chronic wasting disease of captive mule deer: A spongiform encephalopathy. J. Wildl. Dis..

[2] Williams E.S., Young S. (1982). Spongiform encephalopathy of Rocky Mountain elk. J. Wildl. Dis..

[3] Kreeger T.J., Montgomery D.L., Jewell J.E., Schultz W., Williams E.S. (2006). Oral transmission of chronic wasting disease in captive Shira’s moose. J. Wildl. Dis..

[4] Haley N.J., Hoover E.A. (2015). Chronic wasting disease of cervids: Current knowledge and future perspectives. Annu. Rev. Anim. Biosci..

[5] Benestad S.L., Mitchell G., Simmons M., Ytrehus B., Vikøren T. (2016). First case of chronic wasting disease in Europe in a Norwegian free-ranging reindeer. Vet. Res..

[6] Williams E.S., Miller M.W., Kreeger T.J., Kahn R.H., Thorne E.T. (2002). Chronic wasting disease of deer and elk: A review with recommendations for management. J. Wildl. Manag..

[7] Sorensen A., van Beest F.M., Brook R.K. (2014). Impacts of wildlife baiting and supplemental feeding on infectious disease transmission risk: A synthesis of knowledge. Prev. Vet. Med..

[8] Mateus-Pinilla N., Weng H.Y., Ruiz M.O., Shelton P., Novakofski J. (2013). Evaluation of a wild white-tailed deer population management program for controlling chronic wasting disease in Illinois, 2003–2008. Prev. Vet. Med..

[9] Heberlein T.A. (2004). “Fire in the Sistine Chapel”: How Wisconsin responded to chronic wasting disease. Hum. Dimens. Wildl..

[10] Haus J.M., Eyler T.B., Duda M.D., Bowman J.L. (2017). Hunter perceptions toward chronic wasting disease: Implications for harvest and management. Wildl. Soc. Bull..

[11] Miller C.A., Shelby L.B. (2009). Hunters’ general disease risk sensitivity and behaviors associated with chronic wasting disease. Hum. Dimens. Wildl..

[12] Vaske J.J., Timmons N.R., Beaman J., Petchenik J. (2004). Chronic wasting disease in Wisconsin: Hunter behavior, perceived risk, and agency trust. Hum. Dimens. Wildl..

[13] Harper E.E., Miller C.A., Vaske J.J. (2015). Hunter perceptions of risk, social trust, and management of chronic wasting disease in Illinois. Hum. Dimens. Wildl..

[14] Cooney E.E., Holsman R.H. (2010). Influences on hunter support for deer herd reduction as a chronic wasting disease (CWD) management strategy. Hum. Dimens. Wildl..

[15] Gigliotti L.M. (2004). Hunters’ concerns about chronic wasting disease in South Dakota. Hum. Dimens. Wildl..

[16] Needham M.D., Vaske J.J., Manfredo M.J. (2004). Hunters’ behavior and acceptance of management actions related to chronic wasting disease in eight states. Hum. Dimens. Wildl..

[17] Vaske J.J., Miller C.A., Ashbrook A.L., Needham M.D. (2018). Proximity to chronic wasting disease, perceived risk, and social trust in the managing agency. Hum. Dimens. Wildl..

[18] Vaske J.J., Miller C.A. (2018). Hunters and non-hunters chronic wasting disease risk perceptions over time. Soc. Nat. Resour..

[19] Maryland Department of Natural Resources (2016). Chronic Wasting Disease Response Plan.

[20] Maryland Department of Natural Resources (2018). Maryland Annual Deer Report 2017–2018.

[21] West Virginia Department of Natural Resources (2019). West Virginia Hunting and Trapping Regulations Summary July 2019–June 2020.

[22] Pennsylvania Game Commission Positive CWD in Free-Ranging Deer by Township. https://www.pgc.pa.gov/Wildlife/Wildlife-RelatedDiseases/Documents/Positive%20CWD%20in%20Free-Ranging%20Deer%20by%20Township.pdf.

[23] (2018). Responsive Management Maryland Residents’, Landowners’, and Hunters’ Attitudes Toward Deer Hunting and Deer Management.

[24] Cohen J. (1988). Statistical Power Analysis for the Behavioral Sciences.

[25] R Core Team (2019). R: A Language and Environment for Statistical Computing.

[26] Maryland Department of Natural Resources (2009). Maryland Annual Deer Report 2008–2009.

[27] Maryland Department of Natural Resources (2010). Maryland Annual Deer Report 2009–2010.

[28] Maryland Department of Natural Resources (2011). Maryland Annual Deer Report 2010–2011.

[29] Vaske J.J., Miller C.A. (2019). Deer hunters’ disease risk sensitivity over time. Hum. Dimens. Wildl..

[30] Maryland Department of Natural Resources (2012). Maryland Annual Deer Report 2011–2012.

[31] Maryland Department of Natural Resources (2013). Maryland Annual Deer Report 2012–2013.

[32] Maryland Department of Natural Resources (2014). Maryland Annual Deer Report 2013–2014.

[33] Maryland Department of Natural Resources (2015). Maryland Annual Deer Report 2014–2015.

[34] Maryland Department of Natural Resources (2016). Maryland Annual Deer Report 2015–2016.

[35] Maryland Department of Natural Resources (2017). Maryland Annual Deer Report 2016–2017.

[36] Maryland Department of Natural Resources (2019). Maryland Annual Deer Report 2018–2019.

